# Docking and Antiherpetic Activity of 2-Aminobenzo[*de*]-isoquinoline-1,3-diones

**DOI:** 10.3390/molecules20035099

**Published:** 2015-03-19

**Authors:** Rashad Al-Salahi, Ibrahim Alswaidan, Hazem A. Ghabbour, Essam Ezzeldin, Mahmoud Elaasser, Mohamed Marzouk

**Affiliations:** 1 Department of Pharmaceutical Chemistry, College of Pharmacy, King Saud University, P.O. Box 2457, Riyadh 11451, Saudi Arabia; E-Mails: salahi76@yahoo.com (R.A.-S.); ialsuwidan@ksu.edu.sa (I.A.); ghabbourh@yahoo.com (H.A.G.); 2 Drug Bioavailability Lab., College of Pharmacy, King Saud University, P.O. Box 2457, Riyadh 11451, Saudi Arabia; E-Mail: ezzeldin24@hotmail.com; 3 Regional Center for Mycology and Biotechnology, Al-Azhar University, Naser City, Cairo 11759, Egypt; E-Mail: mmelaasser@hotmail.com; 4 Chemistry of Natural Products Group, Center of Excellence for Advanced Sciences, National Research Center, Dokki, Cairo 12622, Egypt

**Keywords:** 2-aminobenzo[*de*]isoquinoline-1,3-dione, docking, HSV-1, HSV-2

## Abstract

As part of our search for new compounds having antiviral effects, the prepared 2-aminonaphthalimide series was examined for its activity against the herpes simplex viruses HSV-1 and HSV-2. This represents the first study of the antiviral effects of this class of compounds. The new series of 2-amino-1*H*-benzo[*de*]isoquinoline-1,3-diones was examined against HSV-1 and HSV-2 using a cytopathic effect inhibition assay. In terms of effective concentration (EC_50_), furaldehyde, thiophene aldehyde and allyl isothiocyanide derivatives **14**‒**16** showed potent activity against HSV-1 (EC_50_ = 19.6, 16.2 and 17.8 μg/mL), compared to acyclovir as a reference drug (EC_50_ = 1.8 μg/mL). Moreover, **14** and **15** were found to exhibit valuable activity against HSV-2. Many of the tested compounds demonstrated weak to moderate EC_50_ values relative to their inactive parent compound (2-amino-1*H*-benzo[*de*]isoquinoline-1,3-dione), while compounds **7**, **9**, **13**, **14**, **15**, **16**, **21** and **22** were the most active set of antiviral compounds throughout this study. The cytotoxicity (CC_50_), EC_50_, and the selectivity index (SI) values were determined. In a molecular docking study, the ligand-receptor interactions of compounds **1**–**24** and their parent with the HSV-1 thymidine kinase active site were investigated using the Molegro Virtual Docker (MVD) software. Based on the potent anti-HSV properties of the previous naphthalimide condensate products, further exploration of this series of 2-amino-1*H*-benzo[*de*]isoquinoline-1,3-diones is warranted.

## 1. Introduction

HSV-1 and HSV-2, as DNA viruses, belong to the alpha herpes virus subfamily, which also includes the varicella zoster virus (VZV). They are common human pathogens and between 60% and 95% of certain populations are infected with HSV-1, and between 6% and 50% with HSV-2 [1,2]. Herpes simplex viruses are common pathogens that also cause herpes genitalis, herpes labialis, encephalitis and keratitis. The infection caused by the two types is mainly transmitted by close personal contact, and the virus establishes lifelong latent infection in the sensory neurons, with recurrent lesions [3,4]. The frequency of HSV-seropositive males is significantly higher in populations infected with the human immunodeficiency virus (HIV). Moreover, sexually transmitted diseases like genital HSV increase the risk of transmission and acquisition of HIV infection [5]; there is also a synergistic relationship between genital herpes and HIV [6]. It was reported that HSV-suppressive therapy greatly reduced genital and plasma levels in patients co-infected by HIV-1 RNA [7]. Hence, reducing the spread of genital herpes can greatly decrease the risk of acquiring or transmitting HIV infection.

Imides of aromatic dicarboxylic acids like naphthalimides are important in the construction of macromolecules as well as in supramolecular assembly. They are useful fluoroprobes for various studies and also serve as precursors for the protection of the amino group [8]. The 1*H*-benzo[*de*]isoquinoline-1,3-diones are also found to play an important role as synthons for the construction of many bioactive compounds such as antitumour and histone deacetylase inhibitors (HDAC) [9,10,11,12,13,14,15,16]. Moreover, they have a high binding affinity towards the 5-HT_1A_ receptor that is expressed in Chinese hamster ovary cells (CHO cells, commonly used in biological, medical research and the most mammalian hosts for industrial production of recombinant protein therapeutics), as determined by fluorescence microscopy [17]. In our recent research, the title compounds were evaluated for their antimicrobial and cytotoxic effects, whereby some of them were found to possess significant activities [18,19]. Despite promising findings in the literature, most such compounds have been poorly studied because of their complicated syntheses [11]. In view of these facts, we aimed to investigate a new prepared series of 2-aminobenzo[*de*]isoquinoline-1,3-diones as antiviral agents against HSV-1 and HSV-2.

## 2. Results and Discussion

### 2.1. Antiviral Activity

**Scheme 1:**
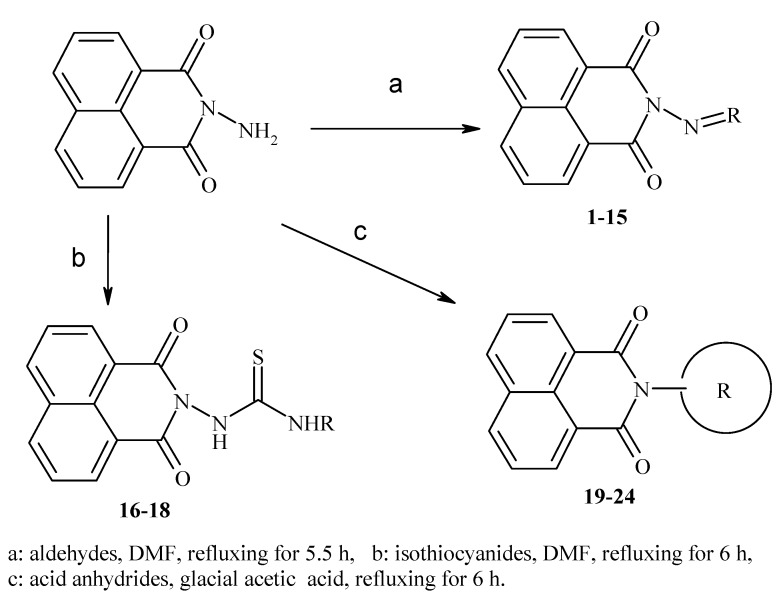
Main routes for synthesis of the target compounds **1**–**24**.

In our previous research, we have described the synthetic routes and full characterisation of compounds **1**–**24**, as illustrated in Scheme 1 and Table 1 [20].

**Table 1:** Synthesised 2-aminobenzo[*de*]isoquinoline-1,3-dione derivatives **1**–**24**.

Cpd.	R	Cpd.	R
**1**	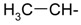	**13**	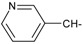
**2**	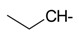	**14**	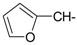
**3**	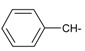	**15**	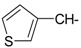
**4**	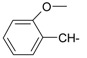	**16**	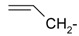
**5**	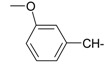	**17**	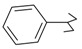
**6**	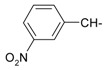	**18**	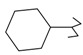
**7**	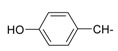	**19**	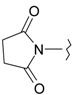
**8**	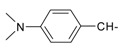	**20**	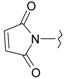
**9**	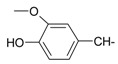	**21**	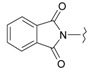
**10**	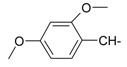	**22**	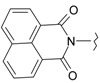
**11**	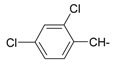	**23**	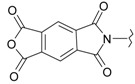
**12**	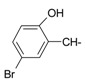	**24**	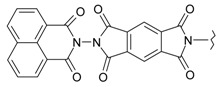

The present work reports our evaluation of the *in vitro* antiviral activity of the newly prepared 2-aminobenzo-[*de*]isoquinoline-1,3-diones against HSV-1 and HSV-2 by means of a cytopathic effect inhibition assay. From the obtained results, it can be seen that the target molecules possess weak to moderate anti-HSV activity. The *in vitro* results of the tested samples against both HSV-1 and HSV-2 are recorded in Table 2, where target compounds **14**, **15** and **16** show remarkable and significant activity against HSV-1, with EC_50_ values of 19.6, 16.2 and 17.8 μg/mL, respectively, with respect to acyclovir (1.8 μg/mL) and their inactive parent compound (2-amino-1*H*-benzo[*de*]isoquinoline-1,3-dione, Scheme 1). Furthermore, **14** and **15** also exhibited good activity against HSV-2, giving EC_50_ values of 71.8 and 56.7 μg/mL, respectively, with respect to acyclovir (3.4 μg/mL). Compounds **7**, **9**, **21** and **22** displayed good activity against HSV-1 (EC_50_ = 113.08, 78.3, 102 and 74.6 μg/mL, respectively) in comparison with the previous active compounds. Additionally, compound **9** demonstrated good effect against HSV-2, relative to **14** and **15**, with an EC_50_ value of 108 μg/mL.

In terms of SI-values, all investigated compounds can be sorted into two groups: inactive (SI < 2) and active (SI ≥ 2) [21]. Accordingly, compounds **4**, **5**, **7**, **9**, **10**, **13**, **16**, **17** and **21**–**23** can be considered as active agents. Compounds **14** and **15** were found to be the most active compounds against HSV-1, and similarly, **9**, **14** and **15** could be accounted the only active compounds against HSV-2 (Table 2).

The present cytotoxicity evaluation of target products **1**–**24** revealed a variety in activity, in which compounds **14**, **15** and **16** yielded the highest effects against HSV-1, while **7**, **9**, **21** and **22** manifested a good antiviral effect. On the other hand, **5**, **6**, **10**, **17**, **18** and **23** showed low activity against HSV-1, while **4**, **7**, **10**, **13** and **21** showed low activity against HSV-2, in comparison with compounds **14**–**16**.

**Table 2:** Antiviral activity of compounds **1**–**24** in terms of CC_50_, EC_50_ (μg/mL) and SI against HSV-1 and HSV-2.

Cpd Nr.	CC_50_	HSV-1		HSV-2	
		EC_50_	SI	EC_50_	SI
**1**	375 ± 25	>500	-	>500	-
**2**	317 ± 13	>500	-	>500	-
**3**	196 ± 22	158 ± 1.8	1.2	>500	-
**4**	486 ± 18	189 ± 3.2	2.6	312 ± 9.6	1.6
**5**	452 ± 9	176 ± 4.6	2.6	>500	-
**6**	316 ± 11	174 ± 1.8	1.8	>500	-
**7**	412 ± 24	113 ± 4.6	3.6	220 ± 3.8	1.9
**8**	289 ± 13	>500	-	>500	-
**9**	236 ± 8	78.3 ± 1.5	3.0	108 ± 4.1	2.2
**10**	380 ± 16	149 ± 5.3	2.6	416 ± 8.2	0.9
**11**	316 ± 24	>500	-	>500	-
**12**	320 ± 18	>500	-	>500	-
**13**	458 ± 27	117.2 ± 3.1	3.9	308 ± 7.1	1.5
**14**	321 ± 23	19.6 ± 0.9	16.4	71.8 ± 1.8	4.5
**15**	180 ± 8	16.2 ± 1.1	11.1	56.7 ± 2.6	3.2
**16**	96 ± 12	17.8 ± 1.4	5.4	292 ± 4.8	0.3
**17**	518 ± 34	236 ± 4.9	2.2	>500	-
**18**	482 ± 14	319 ± 3.7	1.5	>500	-
**19**	220 ± 16	>500	-	>500	-
**20**	220 ± 16	>500	-	>500	-
**21**	490 ± 21	102 ± 5.8	4.8	374 ± 3.7	1.3
**22**	560 ± 46	74.6 ± 3.4	7.5	428 ± 10.2	1.3
**23**	412 ± 18	176 ± 2.4	2.3	>500	-
**24**	386 ± 17	>500	-	>500	-
**Parent**	320 ± 24	>500	-	>500	-
**Acyclovir**	600 ± 18	1.8 ± 0.2	333.33	3.4 ± 0.6	176.47

Notes: Cells treated with DMSO (0.1%) were used as a negative control, and its reading was subtracted from the readings of tested compounds. Parent = 2-amino-1*H*-benzo[*de*]isoquinoline-1,3-dione. Statistics were calculated using one-way ANOVA.

Regarding the elaborated results, biological studies have shown that the type of substituent produced from the condensation reaction with the amino group is a controlling factor governing all of the observed pharmacological properties in the parent structure. This is seen in the increment of antiviral activity in the sequence **15**, **16** and **14** against HSV-1, whereas compound **15** showed higher activity than **14** against HSV-2. This could be attributed to the size and conformation of the heteroaldehyde and isothiocyanide substituents, which could have a substantial effect on the activity and selectivity profiles of such compounds. Throughout this study, we noticed that variation in the substituent on the benzyl ring resulted in a remarkable change in the activity profile. Regarding this fact, it was found that compounds **7** and **9** appeared as the most active among all aromatic aldehydes **3**–**7**, **9** and **10** against HSV-1. In addition, compound **9** seemed to be most highly active against HSV-1 from the group of compounds that also includes **4**, **7** and **10**. Concerning the isothiocyanide derivatives, compound **16** was more active than **17** and **18** against HSV-1. Finally, the antiviral activity increased in the order of **22** > **21** > **23** against HSV-1 and **21** > **22** against HSV-2 in the case of acid anhydride derivatives. In a comparison of EC_50_ with the corresponding CC_50_ values, compounds **1**, **2**, **8**, **11**, **12**, **19**, **20**, **24** and the parent were inactive, whereby they showed higher EC_50_ than CC_50_ values (>500 μg/mL, Table 2). However, compounds **9**, **14**, **15**, **16** and **22** were considered the most active compounds in comparison with others, but less active than acyclovir (Table 2).

The cytopathic effect inhibition assay showed that some of the examined compounds have good antiviral activity against HSV-1 and 2 *in vitro* at non-cytotoxic concentrations with respect to their EC_50_ and SI values, compared to acyclovir. In order to understand the temporal aspects of the antiviral activity of target molecules, it could be suggested that the possible mode of action of such compounds is not the prevention of viral adsorption or penetration, but perhaps an interference in early events of HSV replication, including immediate early (IE) transcriptional events [22,23]. The actual explanation for these changes regarding structure-activity relationship will await the elucidation of the mechanism(s) of action of the compounds.

### 2.2. Molecular Docking

Thymidine kinase (TK) acts catalytically to phosphorylate thymidine, getting it ready for further phosphorylation and eventual incorporation into DNA [24]. So many researches study the binding capabilities TK and other ligands can serve to develop more effective HSV treatment that would act as inhibitors of this enzyme rather than substrates.

**Figure 1:**
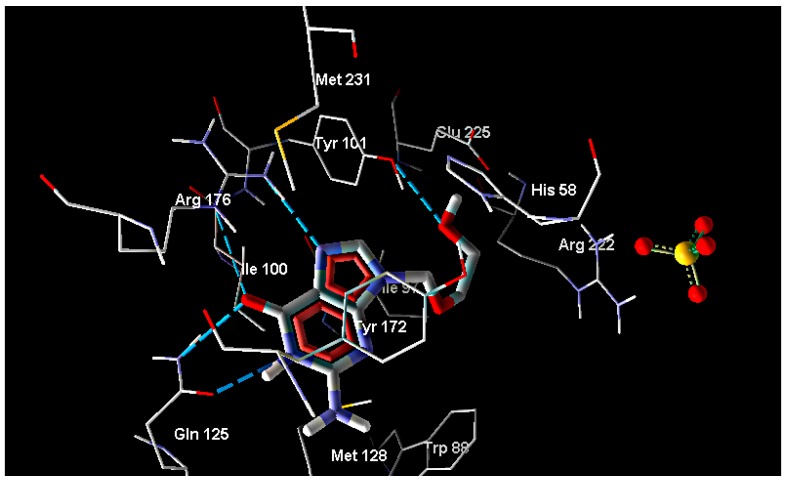
Interaction of acyclovir with thymidine kinase.

**Figure 2:**
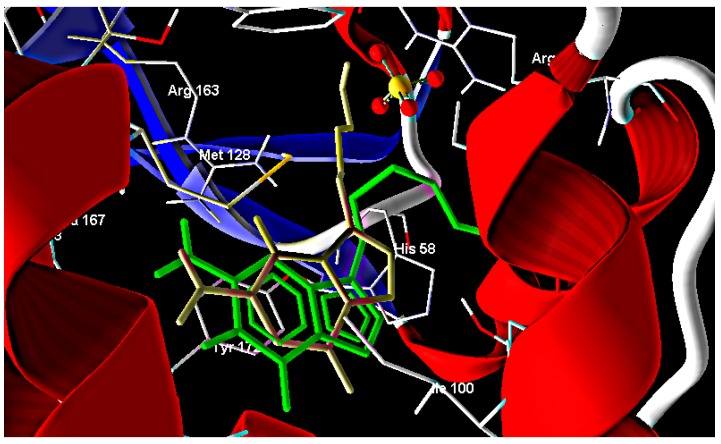
Comparison between the co-crystallized acyclovir (gray) and docked acyclovir (green).

In the present study, the ligand-receptor interactions of compounds **1**–**24** and the parent compound with HSV-1 thymidine kinase active site were investigated by performing docking studies using the Molegro Virtual Docker (MVD) software. The crystal structure of HSV-1 thymidine kinase in complex with acyclovir (PDB code 1KI5) was obtained from the Protein Data Bank [25] (Figure 1). The accuracy of MVD docking protocol was validated and confirmed by docking the co-crystallized acyclovir inside the active site of TK where the docked acyclovir showed little deviation (Figure 2).

Snapshots for the most active tested compounds were taken to reveal their molecular interactions (hydrogen, hydrophobic and/or ionic bonds) with the amino acids-HSV-1 thymidine kinase active sites. MolDock scores between these compounds and receptor were calculated (Table 3).

**Table 3:** MolDock scores of the acyclovir, parent and tested compounds **1**–**24**.

Ligand	MolDock Score	Ligand	MolDock Score	Ligand	MolDock Score
**Parent**	−101.205	**9**	−112.001	**18**	−134.765
**18**	−97.8437	**10**	−108.487	**19**	−112.904
**2**	−102.893	**11**	−104.531	**20**	−118.027
**3**	−102.959	**12**	−115.317	**21**	−93.0316
**4**	−95.8232	**13**	−101.817	**22**	−91.99
**5**	−113.363	**14**	−116.126	**23**	−73.5124
**6**	−127.137	**15**	−112.552	**24**	−133.133
**7**	−109.968	**16**	−129.001	**acyclovir**	−111.737
**8**	−121.392	**17**	−132.879		

**Figure 3:**
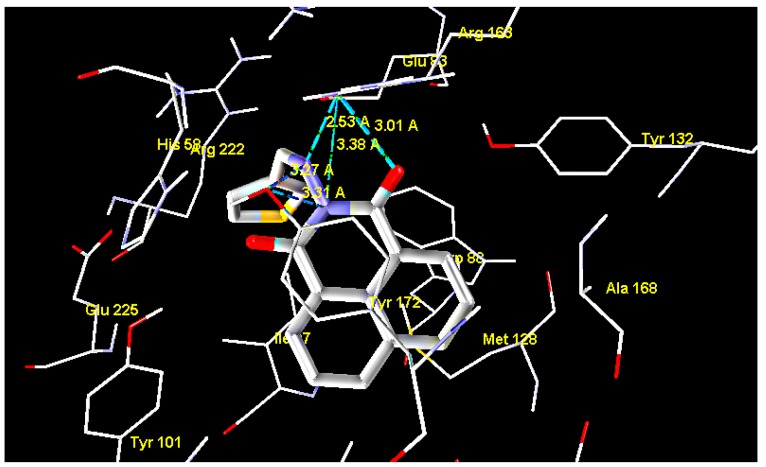
Interaction of compound **15** with thymidine kinase.

The results showed that all tested compounds have affinity for HSV-1 thymidine kinase comparable to that of the reference compound. The docking interaction of the most active compound **15** with the the active site of TK is represented in Figure 3. It is observed that the 2-amino group, carbonyl groups and the nitrogen atom of the benzoisoquinoline-1,3-dione moiety form five H-bond interactions with Glu63 and Tyr172, with bond lengths of 2.53, 3.27, 3.01, 3.38 and 3.31 Å, respectively. In addition, the hydrophobic interactions with Tyr172 alongside the phenyl ring with Tyr132 and Trp88 appear to constrain the molecule in close proximity with the amino acids forming the aforementioned hydrogen bonding [24,25,26].

## 3. Experimental Section

### 3.1. Mammalian Cell Line

Vero cells (derived from the kidney of a normal African green monkey) were obtained from the American Type Culture Collection (ATCC, Manassas, VA, USA). The viral strains used were GHSV-UL46 for HSV-1 and the G strain for HSV-2. The Vero cells were propagated in Dulbecco’s modified Eagle’s medium (DMEM) supplemented with 10% heat-inactivated foetal bovine serum (FBS), 1% L-glutamine, HEPES buffer and 50 µg/mL gentamycin. All cells were maintained at 37 °C in a humidified atmosphere with 5% CO_2_ and were subcultured two times a week [19,27].

### 3.2. Evaluation of the Antiviral Activity

The antiviral screening was performed using a cytopathic effect inhibition assay at the Regional Center for Mycology and Biotechnology (RCMB, Al-Azhar University, Cairo, Egypt) [19,27]. This assay was selected to show specific inhibition of a biological function, that is, a cytopathic effect in susceptible mammalian cells [28]. In brief, monolayers of 10,000 Vero cells adhering at the bottom of the wells in a 96-well microtiter plate were incubated for 24 h at 37 °C in a humidified incubator with 5% CO_2_. The plates were washed with fresh DMEM and challenged with 10^4^ doses of herpes simplex 1 or 2 virus, and then the cultures were simultaneously treated with two-fold serial dilutions of the tested compound, starting from 500 µg/mL and going up to about 2 µg/mL (500, 250, 125, 62.5, …, 1.95 µg/mL) in a fresh maintenance medium; following this, they were incubated at 37 °C for 48 h. An infection control, as well as an untreated Vero cell control were made in the absence of tested compounds. Six wells were used for each concentration of the tested compound. Every 24 h, an observation was made under the inverted microscope until the virus in the control wells showed complete viral-induced cytopathic effects. Antiviral activity was determined by the inhibition of the cytopathic effect compared to a control, that is, the protection offered by the tested compound to the cells was scored [29]. Three independent experiments were assessed, each containing four replicates per treatment. Acyclovir, which is clinically used for the treatment of herpetic viral disease, was used as positive control in this assay system [30].

After the incubation period, the media was aspirated, and then the cells were stained with a 1% crystal violet solution for 30 min. Thereafter, all excess stain was removed by rinsing the plates with tap water. The plates were allowed to dry, and then glacial acetic acid (30%) was added to all wells and mixed thoroughly. The absorbance of the plates was measured after gentle shaking on a microplate reader (Tecan Inc., Morrisville, NC, USA), at 590 nm [19,27]. The viral inhibition rate was calculated as follows:

[(ODtv − ODcv)/(ODcd − ODcv)] × 100%

where ODtv, ODcv and ODcd indicate the absorbance of the tested compounds with virus-infected cells, the absorbance of the virus control and the absorbance of the cell control, respectively.

From these data, the dose that inhibited viral infection by 50% (EC_50_) was estimated with respect to the virus control from the graphic plots, using the STATA modelling software. EC_50_ values were determined directly from the curve obtained by plotting the inhibition of the virus yield against the concentration of the samples. The selectivity index (SI) was calculated from the ratio of CC_50_ to EC_50_ in order to determine whether each compound had sufficient antiviral activity that exceeded its level of toxicity [31]. This index is referred to as a therapeutic index, and it was also used to determine whether a compound warranted further study. Compounds that had an SI-value of 2 or more were considered to be active [19,27].

### 3.3. Cytotoxicity Evaluation Using Viability Assay

The Vero cell lines in the cytotoxicity assay were seeded in 96-well plates at a cell concentration of 1 × 10^4^ cells per well in 100 µL of the growth medium [29]. Fresh medium containing different concentrations of the tested sample was added after 24 h of seeding. Serial two-fold dilutions of the tested compound were added to confluent cell monolayers dispensed into 96-well, flat-bottomed microtiter plates (Falcon, Jersey, NJ, USA) using a multichannel pipette. The microtiter plates were incubated at 37 °C in a humidified incubator with 5% CO_2_ for a period of 48 h. Three wells were used for each concentration of the tested sample. Control cells were incubated without test samples and with or without DMSO. The small percentage of DMSO present in the wells (maximal 0.1%) was not found to affect the experiment [19,27].

After the end of the incubation period, the viable cell yield was determined by a colorimetric method [32,33,34,35,36]. In brief, the process of treatment the media with crystal violet solution and measurement of absorbance at 590 nm using a microplate reader was mentioned above. The absorbance was proportional to the number of surviving cells in the culture plate. All the results were corrected for background absorbance detected in wells without added stain. Treated samples were compared with the cell control in the absence of the tested compounds. All experiments were carried out in triplicate. The cell cytotoxicity effect of each tested compound was calculated [30,37].

### 3.4. Molecular Docking

Molecular docking studies were carried out on a laptop PC, Intel^®^ Core ™ i7-3630 QM CPU @ 2.40 GHz, RAM 8 GB operating under the Windows 7 Professional OS. It consists of several steps; first, the 3D crystal structures of HSV-1 thymidine kinase in complex with acyclovir (PDB code 1KI5) [25] was downloaded from the Brookhaven Protein Data Bank PDB and loaded into Molegro Virtual Docker (MVD 2013.6.0.0 [win32] program fully functional free trial version with time limiting license [38]. All types of atoms, charges and bond hybridization were carefully checked. The MolDock Score [GRID] and MolDock Optimizer routines as implemented in Molegro Virtual Docker (MVD version 2013.6.0.0). The non-bonded oxygen atoms of water molecules, present in the crystal structure, were removed. ChemBio3D Ultra 10 was used to draw the 3D structures of different ligands that were further pre-optimized using free version of Marvinsketch 4.1.13 from Chemaxon Ltd. with MM‏ force field and saved in Tripos mol2 file format [39]. MolDock score functions were used with a 0.3 Å grid resolution. Prior to the calculations of the examined compounds, the MVD software was benchmarked by docking the acyclovir.

### 3.5. Data Analysis

Statistics were done using a one-way ANOVA test [40], and the percentage cell viability was calculated using Microsoft Excel^®^, as follows:

% Cell viability = [(Mean Abs_control_− Mean Abs_test metabolite_)/Mean Abs_control_] × 100

where Abs equals the absorbance at 590 nm. The CC_50_ was estimated from graphic plots and the STATA statistical analysis package was used for the drawing of the dose response curve, in order to calculate CC_50_. Concerning antiviral evaluation, all experiments and data analysis were performed in RCMB, Al-Azhar University, Cairo, Egypt.

## 4. Conclusions

This study has revealed that compounds **14**, **15** and **16** are active agents against both herpes simplex viruses. These compounds could be useful as templates for furthering development and design of more potent antiviral agents.
